# Ag_3_VO_4_ Nanoparticles Decorated Bi_2_O_2_CO_3_ Micro-Flowers: An Efficient Visible-Light-Driven Photocatalyst for the Removal of Toxic Contaminants

**DOI:** 10.3389/fchem.2018.00255

**Published:** 2018-06-27

**Authors:** Shijie Li, Shiwei Hu, Wei Jiang, Yu Liu, Yanping Liu, Yingtang Zhou, Liuye Mo, Jianshe Liu

**Affiliations:** ^1^Key Laboratory of Key Technical Factors in Zhejiang Seafood Health Hazards, Institute of Innovation & Application, Zhejiang Ocean University, Zhoushan, China; ^2^Department of Environmental Engineering, Zhejiang Ocean University, Zhoushan, China; ^3^State Environmental Protection Engineering Center for Pollution Treatment and Control in Textile Industry, College of Environmental Science and Engineering, Donghua University, Shanghai, China

**Keywords:** Ag_3_VO_4_, Bi_2_O_2_CO_3_, heterojunction, visible-light-driven, toxic pollutant removal

## Abstract

Semiconductor-based photocatalysis is of great potential for tackling the environmental pollution. Herein, a novel hierarchical heterostructure of Bi_2_O_2_CO_3_ micro-flowers *in-situ* decorated with Ag_3_VO_4_ nanoparticles was developed by a facile method. Various characterization techniques have been employed to study the physical and chemical property of the novel catalyst. The novel catalyst was utilized for the photocatalytic removal of industrial dyes (rhodamine B, methyl orange) and tetracycline antibiotic under visible-light irradiation. The results indicated that Ag_3_VO_4_/Bi_2_O_2_CO_3_ heterojunctions showed a remarkably enhanced activity, significantly higher than those of bare Ag_3_VO_4_, Bi_2_O_2_CO_3_, and the physical mixture of Ag_3_VO_4_ and Bi_2_O_2_CO_3_ samples. This could be ascribed to an enhanced visible-light harvesting capacity and effective separation of charge carriers by virtue of the construction of hierarchical Ag_3_VO_4_/Bi_2_O_2_CO_3_ heterojunction. Moreover, Ag_3_VO_4_/Bi_2_O_2_CO_3_ also possesses an excellent cycling stability. The outstanding performance of Ag_3_VO_4_/Bi_2_O_2_CO_3_ in removal of toxic pollutants indicates the potential of Ag_3_VO_4_/Bi_2_O_2_CO_3_ in real environmental remediation.

**Highlights**

- Novel architectures of Ag_3_VO_4_ nanoparticles modified Bi_2_O_2_CO_3_ micro-flowers were constructed.
- Novel Ag_3_VO_4_/Bi_2_O_2_CO_3_ exhibited excellent photocatalytic activity and stability.
- Ag_3_VO_4_/Bi_2_O_2_CO_3_ heterojunctions significantly promote the charge separation.

## Introduction

Semiconductor photocatalysis has been regarded as one of the most promising nanotechnologies for the treatment of environmental pollution (Bora and Mewada, 2017; Cates, 2017; Wang W. et al., 2017; Zhang and Ma, 2017; Zhu and Wang, 2017). A significant research topic of photocatalysis is the exploration of highly active photocatalysts (Li et al., 2014, 2017d, 2018b; Adhikari et al., 2015, 2016, 2017; Martin et al., 2015; Zhang et al., 2016, 2017; Mousavi et al., 2018; Yu et al., 2018).

The emergent Bi_2_O_2_CO_3_ has attracted much interest for its good photocatalytic performance in the removal of toxic pollutants (Ni et al., 2016; Yu et al., 2018). However, the photocatalytic properties are still far from satisfactory owing to low solar utilization and fast recombination of electron-hole pairs. To improve the visible-light photocatalytic activity of Bi_2_O_2_CO_3_, various strategies have been developed, such as design of microstructure (Zhao et al., 2011), deposition of metals (Yu et al., 2016), formation of heterojunction (Chen et al., 2016, 2017; Huang et al., 2016; Feng et al., 2017; Hu et al., 2017), and doping with ions (Dong et al., 2014; Xiong et al., 2015). The rational construction of heterojunctions can effectively ameliorate the visible-light absorption ability and significantly suppress the electron-hole recombination (Han et al., 2017; Li et al., 2017b,c,e; Zhong et al., 2018). The further development of novel Bi_2_O_2_CO_3_-based catalysts is still required to offer more potential candidates for practical application and to figure out the reasons for the synergetic effect between the components.

Ag_3_VO_4_, an active VLD photocatalyst, has drawn much attention in virtue of its unique band structures. Ag_3_VO_4_ has been coupled with other semiconductors (e.g., Bi_2_WO_6_ Li et al., 2017a; Zhang and Ma, 2017, BiOI Wang et al., 2015, BiOCl Wang et al., 2016, C_3_N_4_ Wang et al., 2014, BiVO_4_ Yan et al., 2016a, WO_3_ Yan et al., 2016b) to fabricate high-performance photocatalysts. To date, researches on Ag_3_VO_4_ nanoparticles decorated Bi_2_O_2_CO_3_ micro-flowers for the visible-light photo-degradation of toxic contaminants have not been reported.

Herein, we report Ag_3_VO_4_ nanoparticles evenly deposited on the surface of Bi_2_O_2_CO_3_ micro-flowers by a simple precipitation method. Ag_3_VO_4_ nanoparticles can optimize the visible-light response and facilitate the separation of charge carriers, endowing the novel heterojunction with excellent visible-light photocatalytic performance. The plausible visible-light photocatalysis mechanism of Ag_3_VO_4_/Bi_2_O_2_CO_3_ is also proposed.

## Experiment

### Chemicals

Bismuth citrate (BiO_7_C_6_H_5_), sodium carbonate (Na_2_CO_3_), ethanol (CH_3_CH_2_OH), silver nitrate (AgNO_3_), sodium vanadate (Na_3_VO_4_), rhodamine B (RhB), ammonium oxalate (AO), AgNO_3_, tetracycline hydrochloride (TC), *p*-benzoquinone (BQ), methyl orange (MO), and iso-propanol (IPA) were bought from Shanghai Chemical Reagent factory (China). All the reagents were analytic grade and used without further treatment.

### Synthesis of catalysts

Bi_2_O_2_CO_3_ was synthesized via a hydrothermal procedure. Briefly, 2 mmol of sodium carbonate (Na_2_CO_3_) and 2 mmol of bismuth citrate (BiO_7_C_6_H_5_) were sequentially dissolved in the solution containing 30 mL of deionized water and 5 mL of absolute ethanol with the assistance of ultra-sonication. The resulting solution was sealed in a 50 mL autoclave and heated at 160°C for 25 h. After the reactor system was cooled down, the precipitants were washed thoroughly with de-ionized water and dried at 80°C overnight.

Ag_3_VO_4_/Bi_2_O_2_CO_3_ heterojunctions were constructed by a simple precipitation method. Briefly, an appropriate amount of Bi_2_O_2_CO_3_ was ultrasonically suspended in 50 mL of H_2_O. Then, 3 mmol AgNO_3_ was dissolved in the above solution under magnetical stirring. After that, Na_3_VO_4_ (20 mL, 0.05 mol L^−1^) solution was slowly dropped into the mixture with vigorous stirring for 5 h. Lastly, the obtained solids were washed with deionized water four times and dried at 80°C for 10 h to get the Ag_3_VO_4_/Bi_2_O_2_CO_3_ heterojunctions. The heterojunctions with different Bi_2_O_2_CO_3_/Ag_3_VO_4_ weight ratios of 0.05/1, 0.10/1, 0.30/1, and 0.50/1 are labeled as AVO/BOC-5, AVO/BOC-10, AVO/BOC-30, and AVO/BOC-50, respectively. Ag_3_VO_4_ was prepared in the absence of Bi_2_O_2_CO_3_.

### Characterization of catalysts

The scanning electron microscopy (SEM, Hitachi S-4800) and transmission electron microscopy (TEM, JEM-2100 JEOL) were applied to characterize the morphology of the samples. Bruker Quantax 400 energy-dispersive X-ray spectroscopy (EDS) was used to identify the chemical composition. Powder X-ray diffractometer (XRD, MSAL XD2) was used to get the XRD patterns of the samples. UV–Vis diffuse reflectance spectra (DRS) were obtained on a spectrophotometer (Shimadzu UV−2600). Photoluminescence (PL) spectra of the samples were recorded on a Hitachi RF-6000 spectrophotometer.

### Photocatalytic tests

Pollutant [rhodamine B (RhB), methyl orange (MO), and tetracycline hydrochloride (TC)] removal performances were tested under visible-light irradiation, 300 W xenon lamp with filter (λ > 400 nm). The photocatalytic reaction was conducted in a glass reactor containing 80 mL of RhB (5 mg L^−1^), MO (5 mg L^−1^), or TC (20 mg L^−1^) solution, and 40 mg of catalyst. The solution was first ultrasonically dispersed for 1 min and then magnetically stirred in the dark for 1 h. 1.5 mL of solution was taken at specified time, and centrifuged to remove the solids. The pollutant concentrations were determined using a Shimadzu UV-2600 spectrophotometer. Total organic carbon (TOC) value of the pollutant solutions during reaction was detected on a Shimadzu TOC analyzer.

## Results and discussion

### Characterization

**Figure 1:**
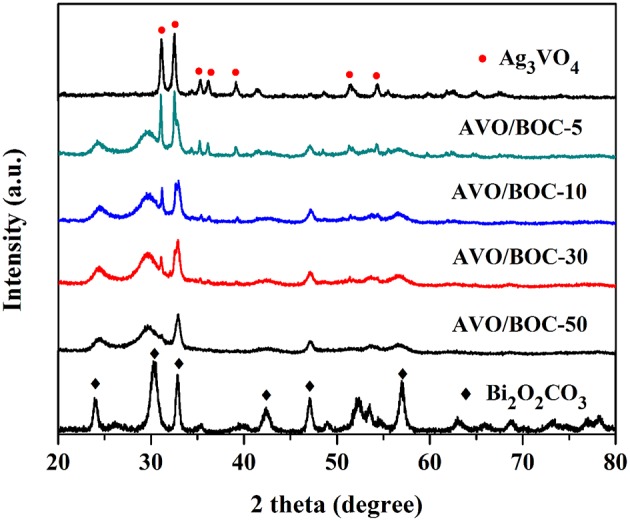
XRD patterns of Ag_3_VO_4_/Bi_2_O_2_CO_3_ heterojunctions (AVO/BOC-5, AVO/BOC-10, AVO/BOC-30, and AVO/BOC-50), pure Bi_2_O_2_CO_3_, and Ag_3_VO_4_.

Figure 1 displays the XRD patterns of the as-prepared pure Ag_3_VO_4_, Bi_2_O_2_CO_3_, and their heterojunctions (AVO/BOC-5, AVO/BOC-10, AVO/BOC-30, and AVO/BOC-50). The diffraction peaks of Ag_3_VO_4_ and Bi_2_O_2_CO_3_ prepared match well with monoclinic phase of Ag_3_VO_4_ (JCPDS 43-0542) and tetragonal phase of Bi_2_O_2_CO_3_ (JCPDS 41-1488), respectively.

When a small amount of Ag_3_VO_4_ was introduced, no diffraction peaks of Ag_3_VO_4_ can be observed in the XRD pattern of AVO/BOC-50. As the Ag_3_VO_4_ content increases, AVO/BOC-30, AVO/BOC-10, and AVO/BOC-5 show the diffraction peaks of both Bi_2_O_2_CO_3_ and Ag_3_VO_4_, indicating the successful fabrication of Ag_3_VO_4_/Bi_2_O_2_CO_3_ heterojunctions.

**Figure 2:**
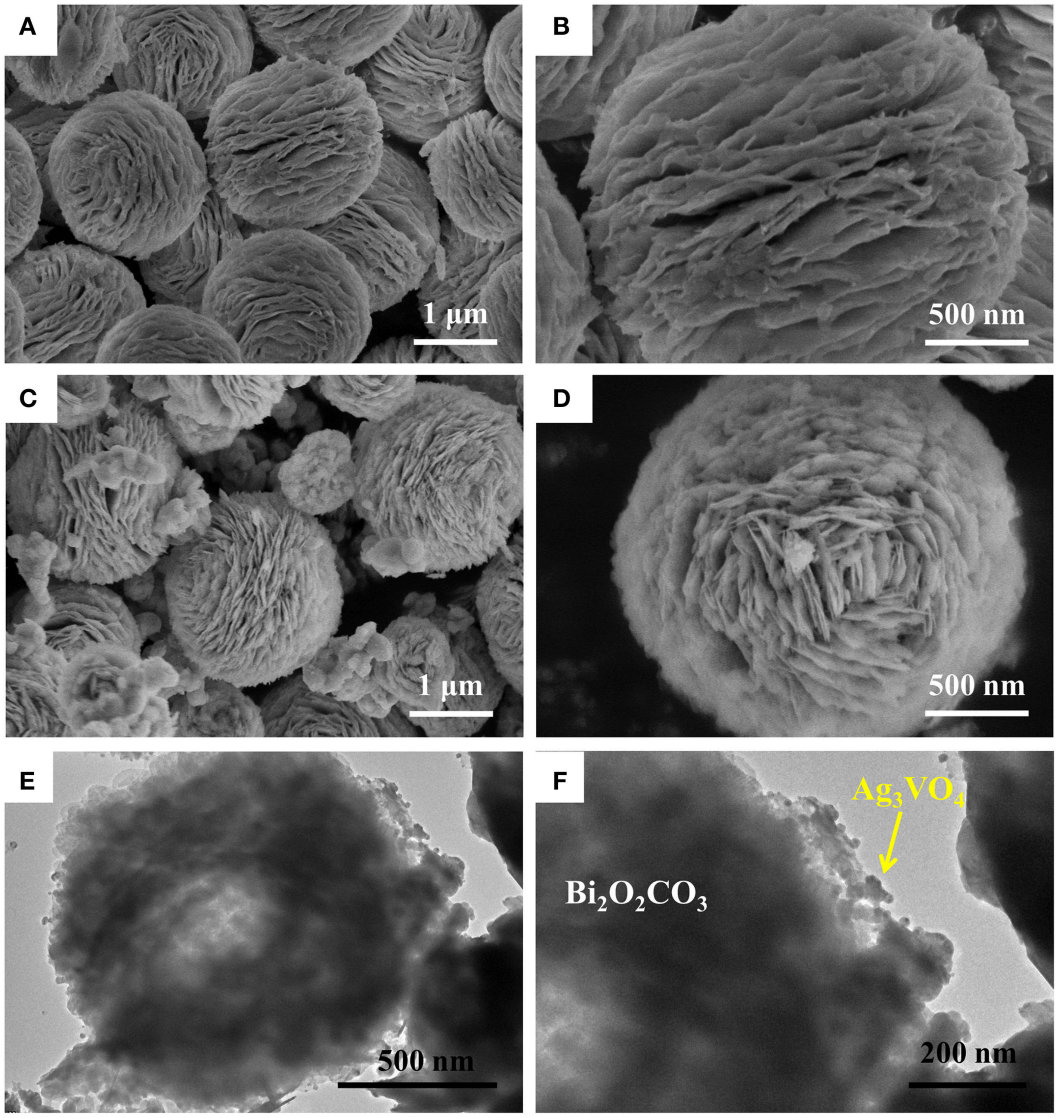
SEM images of pristine Bi_2_O_2_CO_3_ **(A,B)** and AVO/BOC-30 **(C,D)**; TEM images of AVO/BOC-10 **(E,F)**.

The microstructures of Bi_2_O_2_CO_3_ and Ag_3_VO_4_/Bi_2_O_2_CO_3_ heterojunctions were investigated by using SEM. The SEM images in Figures 2A,B show that the obtained Bi_2_O_2_CO_3_ exhibits flower-like microspheres constructed by countless nano-plates (Zhao et al., 2011). The SEM images in Figures 2C,D show the representative AVO/BOC-10 also possesses sphere-like morphology as that for pure Bi_2_O_2_CO_3_. Of note, numerous Ag_3_VO_4_ nanoparticles were deposited on the surfaces of AVO/BOC-10.

The more detailed microstructures of AVO/BOC-10 were studied by TEM. As shown in Figures 2E,F, AVO/BOC-10 consists of Bi_2_O_2_CO_3_ micro-flower (diameter: ~1.1 μm) and Ag_3_VO_4_ nanoparticles (size: ~26 nm), and they were tightly combined with each other to generate closely hybrid hetero-structure, in favor of transfer and separation of charge carriers (Huang et al., 2015; Zhang et al., 2016; Li et al., 2017a).

**Figure 3:**
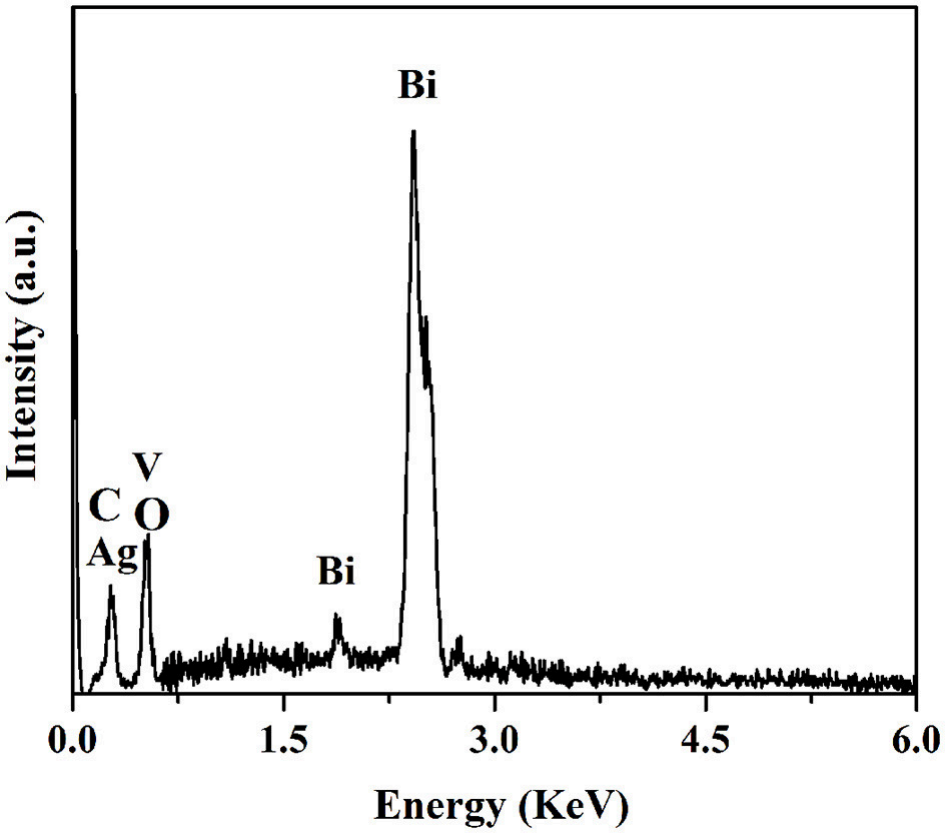
EDS spectra of AVO/BOC-10.

The corresponding EDS spectra of AVO/BOC-10 revealed that only signals for Ag, V, Bi, C, and O elements were detected, indicating the high purity of the sample (Figure 3). The above results verified that the facile precipitation method could successfully fabricate Ag_3_VO_4_/Bi_2_O_2_CO_3_ heterojunctions with intimate contact between two constituents.

**Figure 4:**
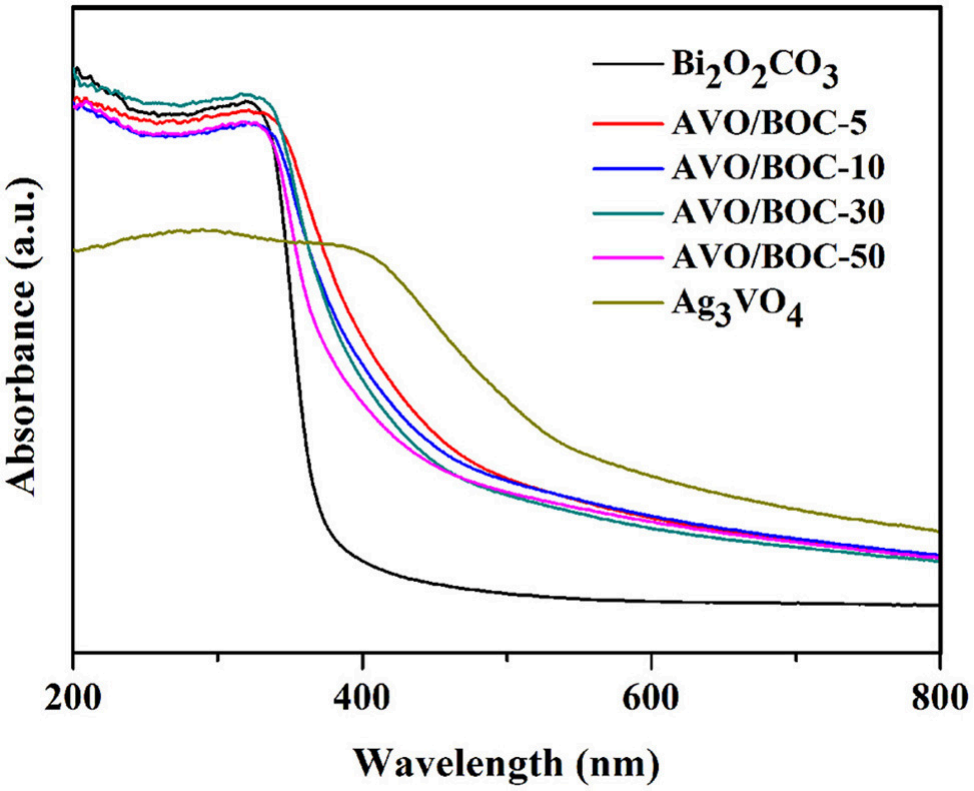
The UV–Vis DRS of bare Bi_2_O_2_CO_3_, Ag_3_VO_4_ and Ag_3_VO_4_/Bi_2_O_2_CO_3_ heterojunctions.

The sunlight absorption capability and band structures of a photocatalyst usually exert a significant effect on its photocatalytic performance. Thus, the UV–Vis DRS spectra of bare Bi_2_O_2_CO_3_, Ag_3_VO_4_, and Ag_3_VO_4_/Bi_2_O_2_CO_3_ heterojunctions are measured and illustrated in Figure 4. Bi_2_O_2_CO_3_ exhibited a strong absorption in the UV region with the absorption edge at 385 nm, in accordance with the previous reports (Zhao et al., 2011; Hu et al., 2017). Ag_3_VO_4_ performed a 575 nm absorption edge in the VL region, consistent with the reported values (Wang et al., 2015; Yan et al., 2016b; Li et al., 2017a). Intriguingly, the combination of Ag_3_VO_4_ and Bi_2_O_2_CO_3_ substantially ameliorated the VL absorption properties of the heterojunctions, which is beneficial for the effective utilization of solar energy.

The bandgap width (Eg) was estimated according to the equation: Eg = 1240/λg (eV), and the Eg value of Ag_3_VO_4_ and Bi_2_O_2_CO_3_ are about 2.15 and 3.23 eV. The band positions (*E*_VB_ and *E*_CB_) of Ag_3_VO_4_ and Bi_2_O_2_CO_3_ can be calculated using the following formula:

(1) $$\begin{array}{r} {\text{E}}_{\text{VB}}=\text{X}-{\text{E}}^{\text{e}}+0.5{\text{E}}_{\text{g}} \end{array}$$

(2) $$\begin{array}{r} {\text{E}}_{\text{CB}}={\text{E}}_{\text{VB}}-{\text{E}}_{\text{g}} \end{array}$$

Where *X* [ca. 6.36 eV for Ag_3_VO_4_ (Li et al., 2017a), and ca. 6.54 eV for Bi_2_O_2_CO_3_ Liang et al., 2014] is the electronegativity of the semiconductor. *E*^e^ value equals to ~4.5 eV. On the basis of above data, the *E*_VB_ and *E*_CB_ of Ag_3_VO_4_ were determined as 0.01 and 2.14 eV, while those of Bi_2_O_2_CO_3_ were 0.2 and 3.53 eV.

### Photocatalytic property

**Figure 5:**
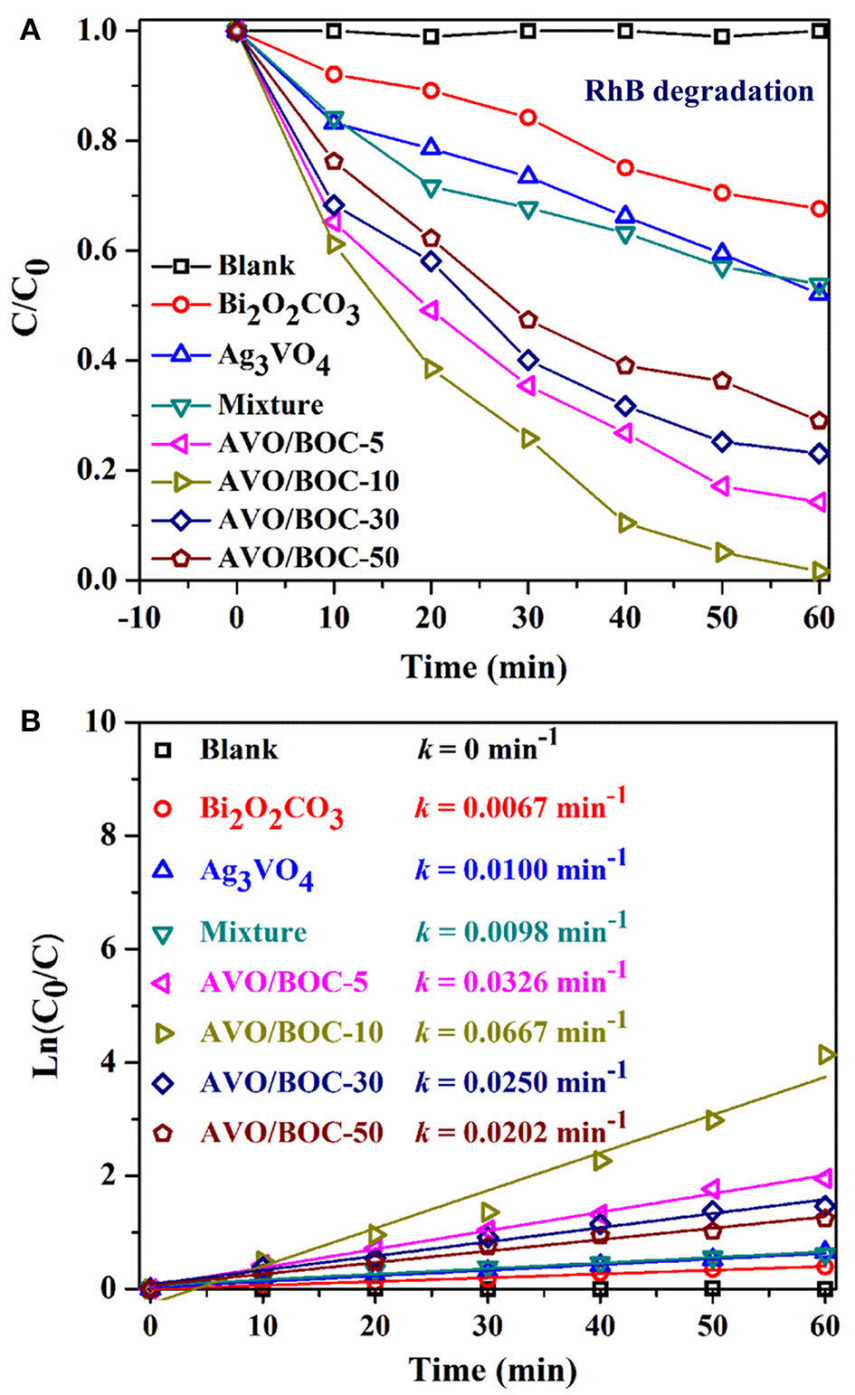
**(A)** Photocatalytic degradation efficiency of RhB over different catalysts under visible light. **(B)** Rate constants of RhB degradation during reaction over different catalysts.

**Figure 6:**
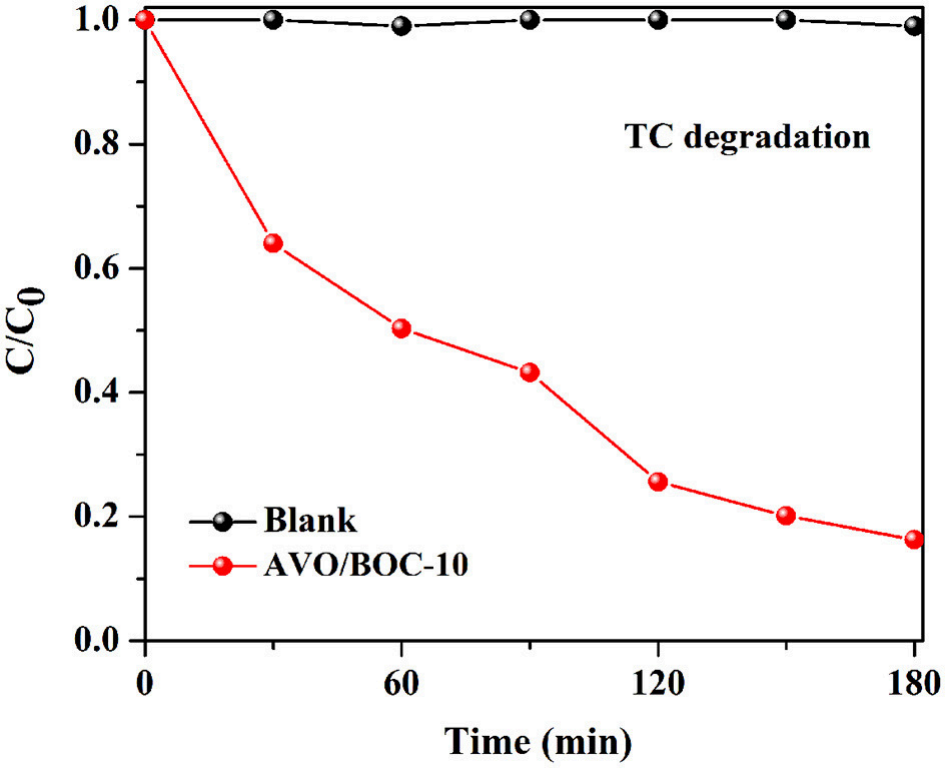
Photocatalytic degradation efficiency of TC over AVO/BOC-10.

The VLD photocatalytic activity of Ag_3_VO_4_/Bi_2_O_2_CO_3_ heterojunctions was studied through the degradation of RhB (Figure 5), MO (Figure S1), and TC (Figure 6). Figure 5A displays the concentration change of RhB dye solution under visible light with the as-prepared catalysts. The blank test conducted without the presence of catalyst showed that RhB was not degraded after 60 min of irradiation. The photocatalytic activity of pristine Bi_2_O_2_CO_3_ is much lower than other samples and the RhB degradation efficiency is 31.4%, mainly due to its large bandgap (Yu et al., 2016). Only 49.8% of RhB was removed by pure Ag_3_VO_4_ due to the high recombination rate of charge carriers (Yan et al., 2016b). Inspiringly, when Bi_2_O_2_CO_3_ was decorated with Ag_3_VO_4_, the catalytic activity of these heterojunctions was substantially improved. After 60 min of irradiation, the RhB degradation efficiencies by using AVO/BOC-5, AVO/BOC-10, AVO/BOC-30 and AVO/BOC-50 were 85.8, 98.4, 76.9, and 71.1%, respectively, much higher than that by using the pristine Bi_2_O_2_CO_3_, Ag_3_VO_4_, or the mechanical mixture (91 wt% Ag_3_VO_4_ + 9 wt% Bi_2_O_2_CO_3_). The activity of Ag_3_VO_4_/Bi_2_O_2_CO_3_ increases gradually and then declines regularly, while AVO/BOC-10 has the highest photocatalytic activity, indicating the vital role of Ag_3_VO_4_ in enhancing the activity.

The degradation rate constants (k) of RhB were presented in Figure 5B. The photocatalytic activity of AVO/BOC-10 achieved the maximum value of k = 0.0667 min^−1^, it was about 8.9, 5.7, and 5.8-folds higher than pure Bi_2_O_2_CO_3_ (0.0067 min^−1^), Ag_3_VO_4_ (0.0100 min^−1^), and the mechanical mixture (0.0098 min^−1^).

The degradation of antibiotic TC or industrial dye MO was also performed to further test the VL photocatalysis of AVO/BOC-10 (Figure 6 and Figure S1). Apparently, AVO/BOC-10 also showed high activity in the degradation of TC and MO (Figure S1). The TC or MO degradation efficiency with AVO/BOC-10 as the catalyst was 83.7 or 94.2% after 180 min of reaction. These results demonstrate that AVO/BOC-10 exhibits extraordinary photocatalytic activity in the removal of toxic pollutants.

**Figure 7:**
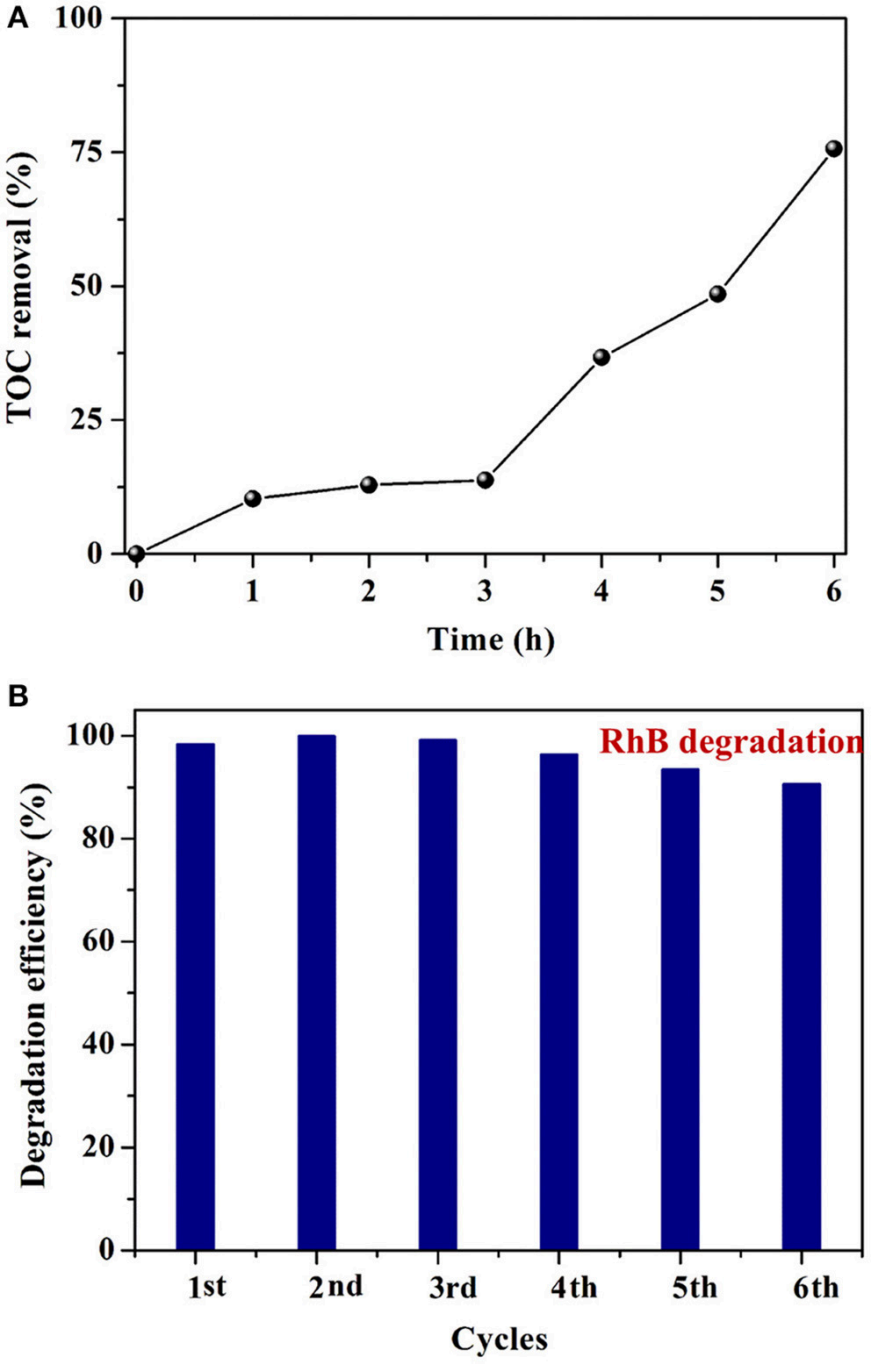
**(A)** TOC removal rate during RhB degradation with AVO/BOC-10; **(B)** The cycling performance of AVO/BOC-10.

To assess the mineralization capability of Ag_3_VO_4_/Bi_2_O_2_CO_3_, the TOC data during RhB (50 mg L^−1^) degradation over AVO/BOC-10 (200 mg) was recorded and analyzed (Figure 7A). It is found that the TOC removal efficiency of RhB with AVO/BOC-10 is 76.4% after 6 h of reaction, suggesting that AVO/BOC-10 possesses strong mineralization ability during the photocatalytic reaction.

For evaluating the stability of Ag_3_VO_4_/Bi_2_O_2_CO_3_, six successive cycles of RhB degradation with AVO/BOC-10 as a catalyst were carried out. Inspiringly, no apparent loss of activity of the catalyst in six times of reuse was observed, and the RhB removal efficiency retained 92.1% in the sixth run (Figure 7B). In addition, the catalyst before and after six runs was characterized by XRD technique (Figure S2), and no obvious changes in the crystalline phases was detected, verifying the good stability of AVO/BOC-10. Moreover, the cycling degradation involving TC antibiotic further confirms the good stability of AVO/BOC-10 (Figure S3). The photocatalytic tests demonstrate that AVO/BOC-10 endowed with high activity and stability is a kind of promising VLD photocatalysts, exhibiting great potential for wastewater treatment.

### Origin of the improved performance

**Figure 8:**
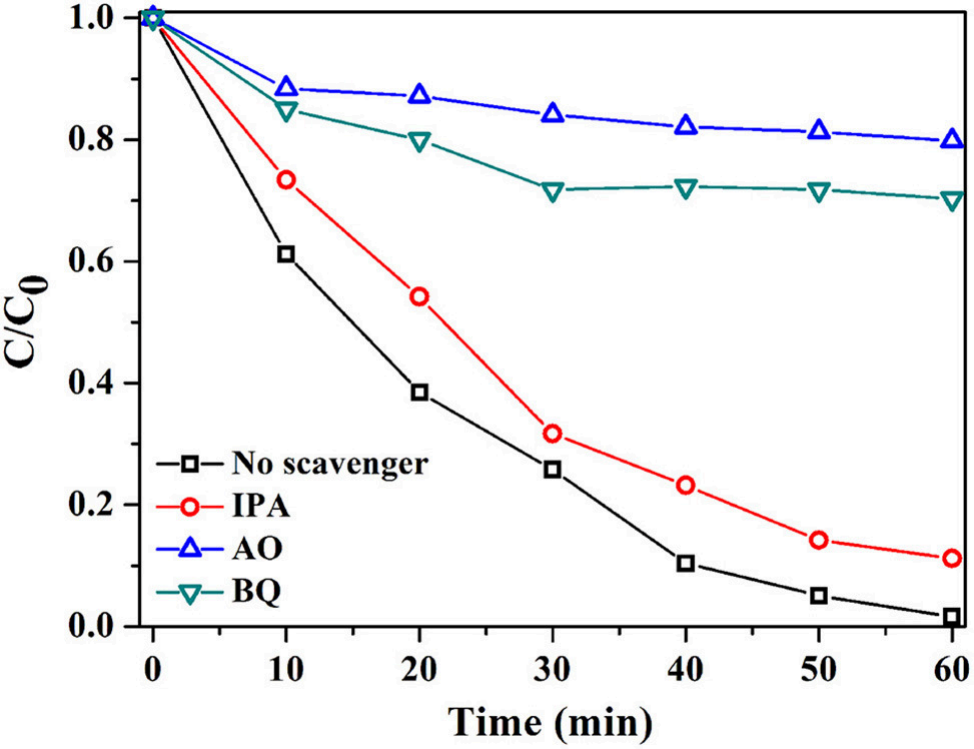
PL spectra of pristine Bi_2_O_2_CO_3_ and AVO/BOC-10.

The photocatalytic activity depends strongly on the separation efficiency of photo-induced charge carriers (Hu et al., 2017; Li et al., 2017a), thus, photoluminescence (PL) spectrum of the samples were acquired to illustrate the electron-hole separation (Figure 8). Apparently, the PL intensity of AVO/BOC-10 is much weaker than that of pristine Bi_2_O_2_CO_3_. Since a weaker PL intensity signifies higher separation rate of charge carriers, AVO/BOC-10 should possess a higher separation efficiency compared with Bi_2_O_2_CO_3_. That is to say, the photo-induced electron-hole pairs were efficiently separated in AVO/BOC-10 system due to the interfacial charge transfer, resulting in the elevated photocatalytic activity.

**Figure 9:**
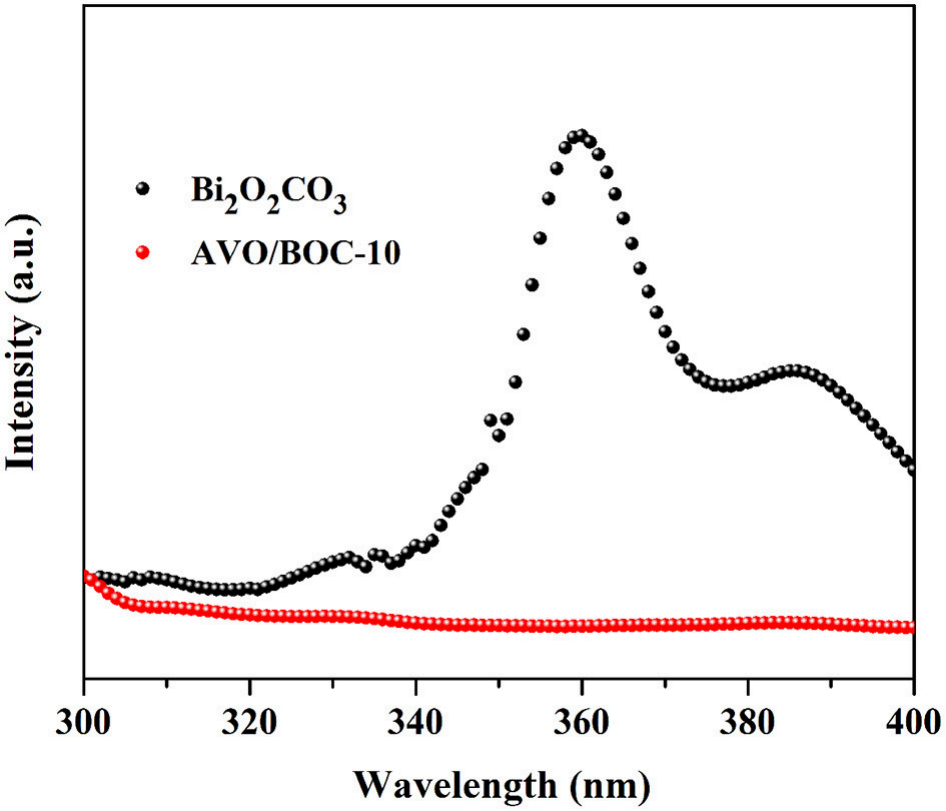
Radical scavenge tests with AVO/BOC-10 as the catalyst.

For mechanistic study, various additives were employed to research the main active species in the photocatalytic reaction process (Figure 9) (Zhang and Ma, 2017; Li et al., 2018). When 1 mmol of BQ (benzoquinone, O${}_{2}^{∙-}$ scavenger) or AO (ammonium oxalate, h^+^ scavenger) was introduced, the activity of AVO/BOC-10 was substantially quenched, and the RhB degradation efficiency declined from 98.4 to 29.7% or 20.2%, revealing that O${}_{2}^{∙-}$ and h^+^ should play vital roles in the photocatalysis. On the contrary, no obvious decrease in the activity was observed with adding 1 mmol of IPA (isopropyl alcohol, ∙OH scavenger), signifying that ∙OH plays a minor role.

**Figure 10:**
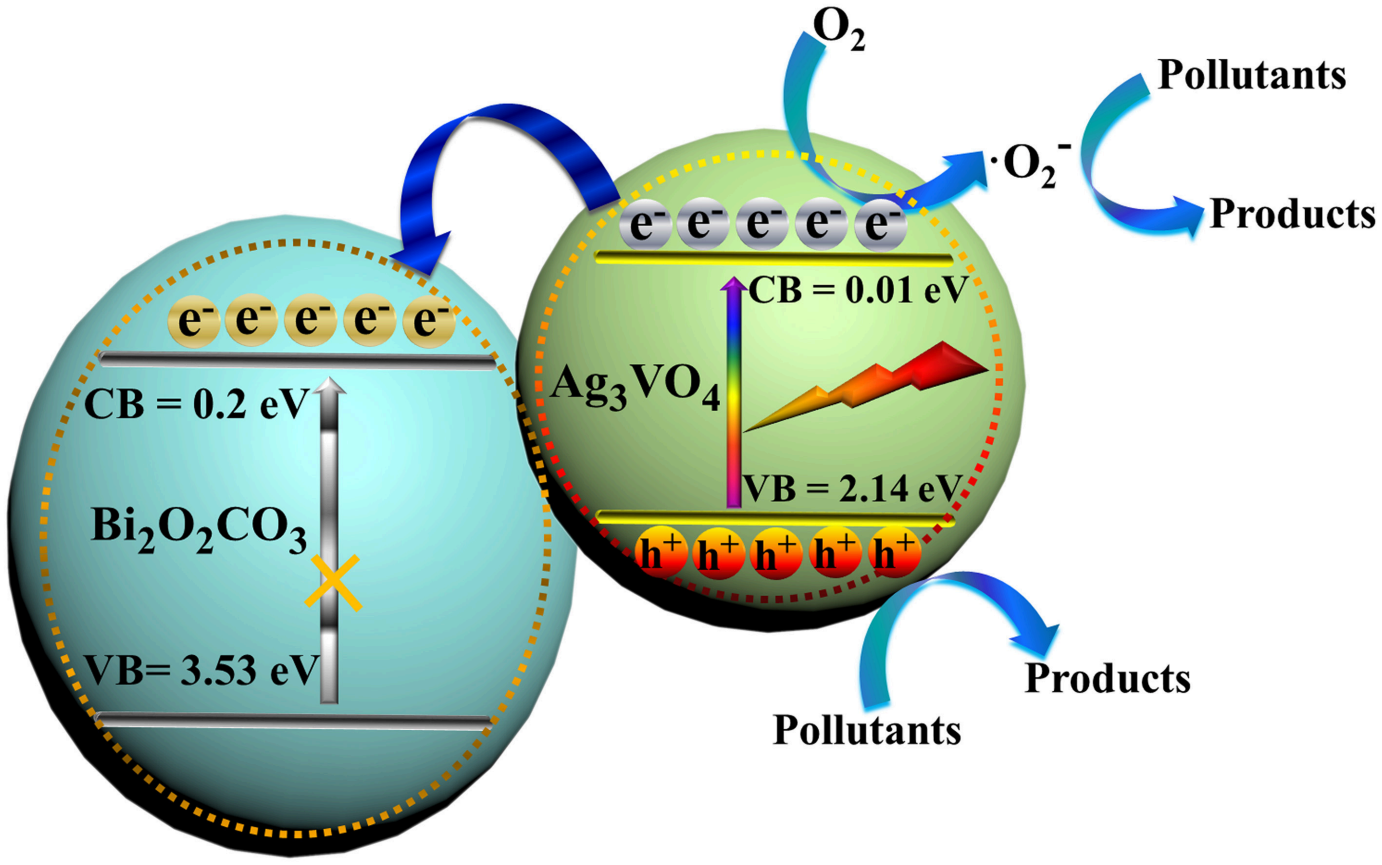
Photocatalytic degradation mechanism of toxic pollutant over Ag_3_VO_4_/Bi_2_O_2_CO_3_.

Based on this systematic investigation, a possible visible-light photocatalytic mechanism for pollutant degradation over Ag_3_VO_4_/Bi_2_O_2_CO_3_ is proposed (Figure 10). Apparently, the position of CB and VB between Ag_3_VO_4_ and Bi_2_O_2_CO_3_ are beneficial to achieve effective separation of photo-generated charge carriers (Wang F. F. et al., 2017; Ye et al., 2018). Photo-generated electrons and holes are produced on Ag_3_VO_4_ under visible-light illumination. Since the CB of Bi_2_O_2_CO_3_ is lower than that of Ag_3_VO_4_, the electrons can be injected readily from Ag_3_VO_4_ to Bi_2_O_2_CO_3_. The electrons on the CB of Bi_2_O_2_CO_3_ can react with O_2_ to form active O${}_{2}^{∙-}$, degrading toxic pollutants such as RhB/MO/TC. Simultaneously, the holes with strong oxide capability in the VB of Ag_3_VO_4_ are available to take part in the decomposition of pollutants. In such a way, the electrons and holes can be effectively utilized, as evidenced by the result of PL spectra of Bi_2_O_2_CO_3_ and Ag_3_VO_4_/Bi_2_O_2_CO_3_ (Figure 8). In summary, the combination of Ag_3_VO_4_ and Bi_2_O_2_CO_3_ enhances the charge separation, leading to the high activity.

## Conclusions

In this study, innovative Ag_3_VO_4_/Bi_2_O_2_CO_3_ heterojunction photocatalysts were synthesized *via* a simple procedure. The Ag_3_VO_4_/Bi_2_O_2_CO_3_ heterojunction (AVO/BOC-10) displayed the optimal photocatalytic properties toward the degradation of toxic pollutants (RhB dye, MO dye, and TC antibiotic), much higher than pristine Bi_2_O_2_CO_3_ and Ag_3_VO_4_. The close contact and the match of bandgap structure between both constituents boost the separation of electron-hole pairs, mainly accounting for the activity enhancement. The holes and O${}_{2}^{∙-}$ were determined as the primary active species responsible for the efficient removal and mineralization of the toxic pollutants. Therefore, Ag_3_VO_4_/Bi_2_O_2_CO_3_ holds huge potential for real wastewater treatment.

## Author contributions

SL designed and performed the experiments, and data analysis. SH, WJ, YuL, YaL, YZ, JL, and LM assisted with some of the tests. SL wrote the main content of the paper. All authors have read and approved the paper to be submitted.

### Conflict of interest statement

The authors declare that the research was conducted in the absence of any commercial or financial relationships that could be construed as a potential conflict of interest.
