# Supplementary material for: Ag3VO4 Nanoparticles Decorated Bi2O2CO3 Micro-Flowers: An Efficient Visible-Light-Driven Photocatalyst for the Removal of Toxic Contaminants
Source: Front Chem. 2018 Jun 27;6:255. doi: 10.3389/fchem.2018.00255 (PMC6036280; doi:10.3389/fchem.2018.00255)
Supplement: Supplementary file 1 [file Data_Sheet_1.DOC]

**Supplementary Data**

**Ag_3_VO_4_ nanoparticles decorated Bi_2_O_2_CO_3_ micro-flowers: An efficient visible-light-driven photocatalyst for the removal of toxic contaminants**

Shijie Li^1*^, Shiwei Hu^1^, Wei Jiang^1^, Yu Liu^1^, Yanping Liu^2^, Yingtang Zhou^1*^, Liuye Mo^1^, Jianshe Liu^3^

^1^ Key Laboratory of key technical factors in Zhejiang seafood health hazards, Institute of Innovation & Application, Zhejiang Ocean University, Zhoushan, Zhejiang Province, 316022, China.

^2^ Department of Environmental Engineering, Zhejiang Ocean University, Zhoushan, Zhejiang Province, 316022, China;

^3^ State Environmental Protection Engineering Center for Pollution Treatment and Control in Textile Industry, College of Environmental Science and Engineering, Donghua University, Shanghai 201620, China.

* Email address: [lishijie@zjou.edu.cn](mailto:lishijie@zjou.edu.cn) (Shijie Li); [zhouyingtang@zjou.edu.cn](mailto:zhouyingtang@zjou.edu.cn) (Yingtang Zhou)

**Figures**

**
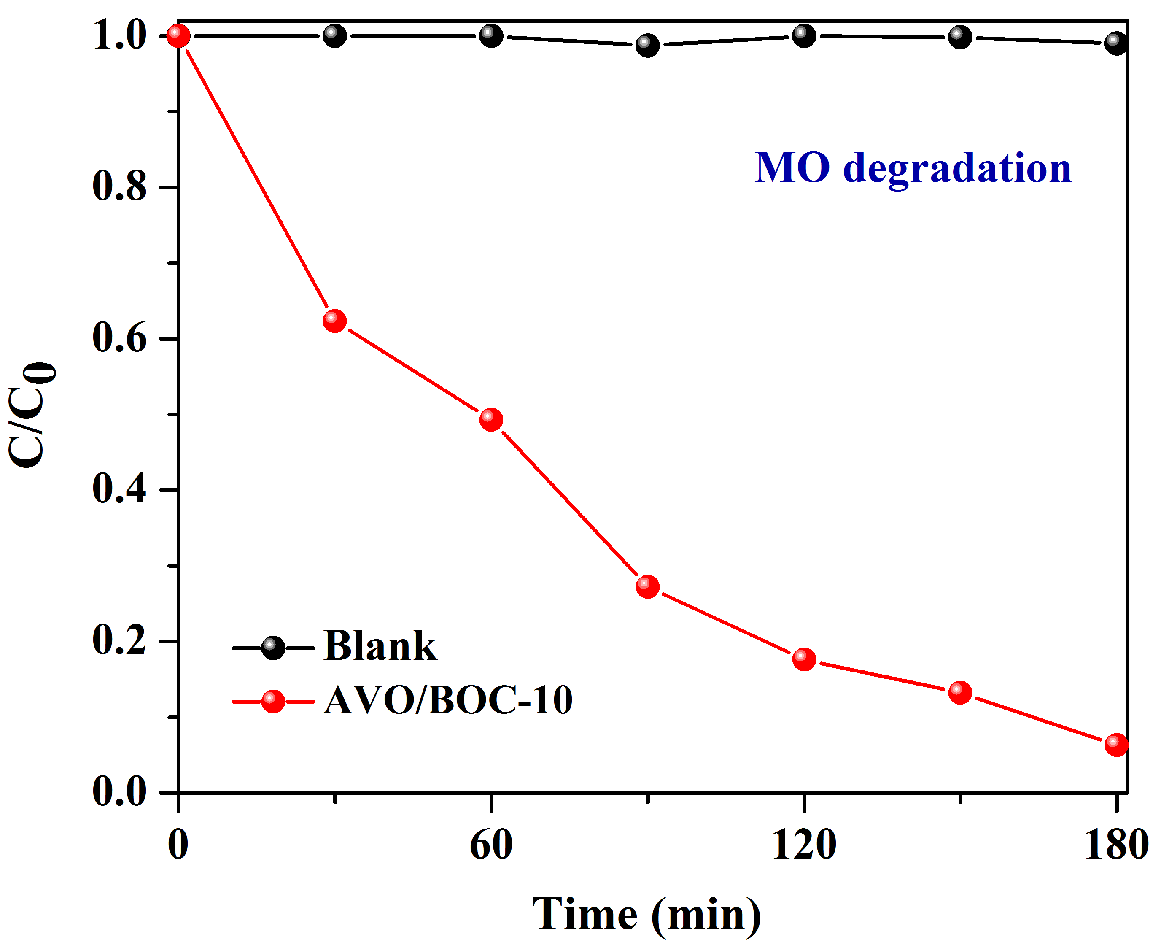
**

**Figure S1.** Photocatalytic degradation efficiency of MO over AVO/BOC-10.

**
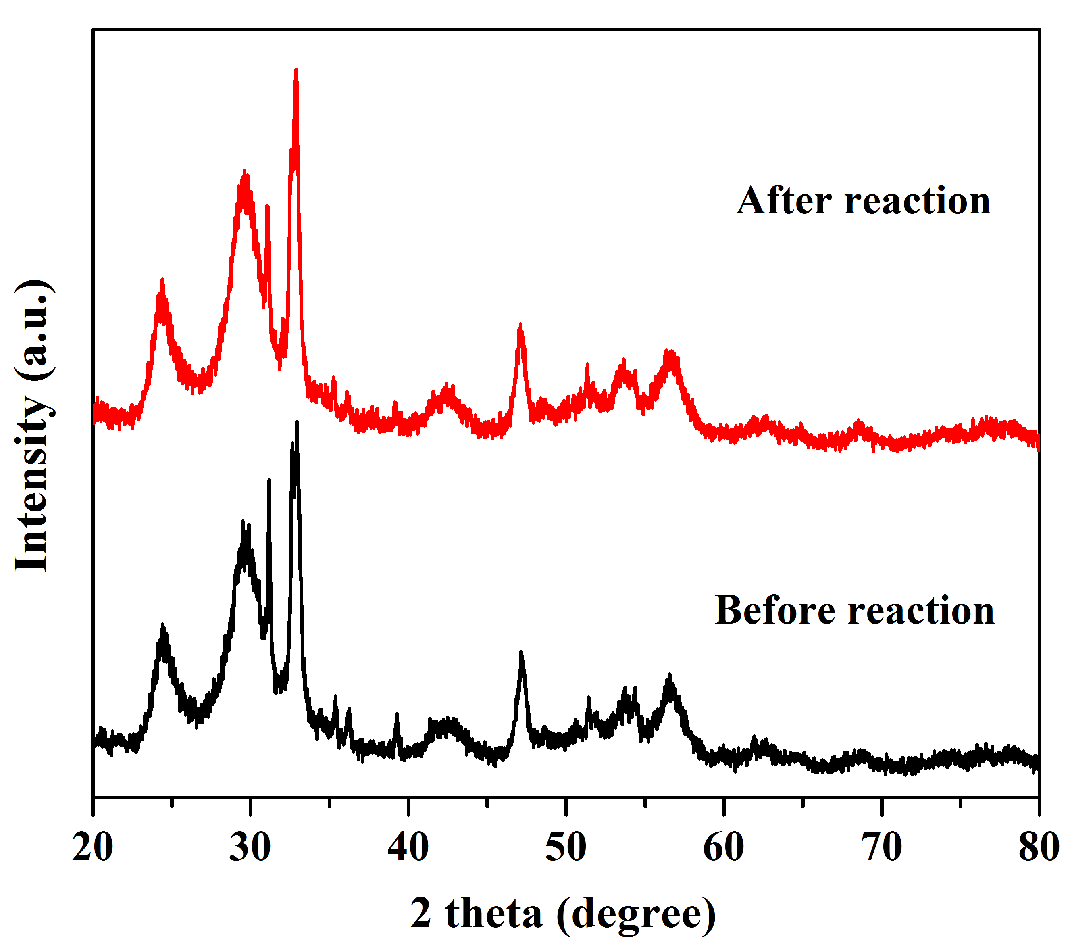
**

**Figure S2.** The XRD patterns of the fresh and used AVO/BOC-10.


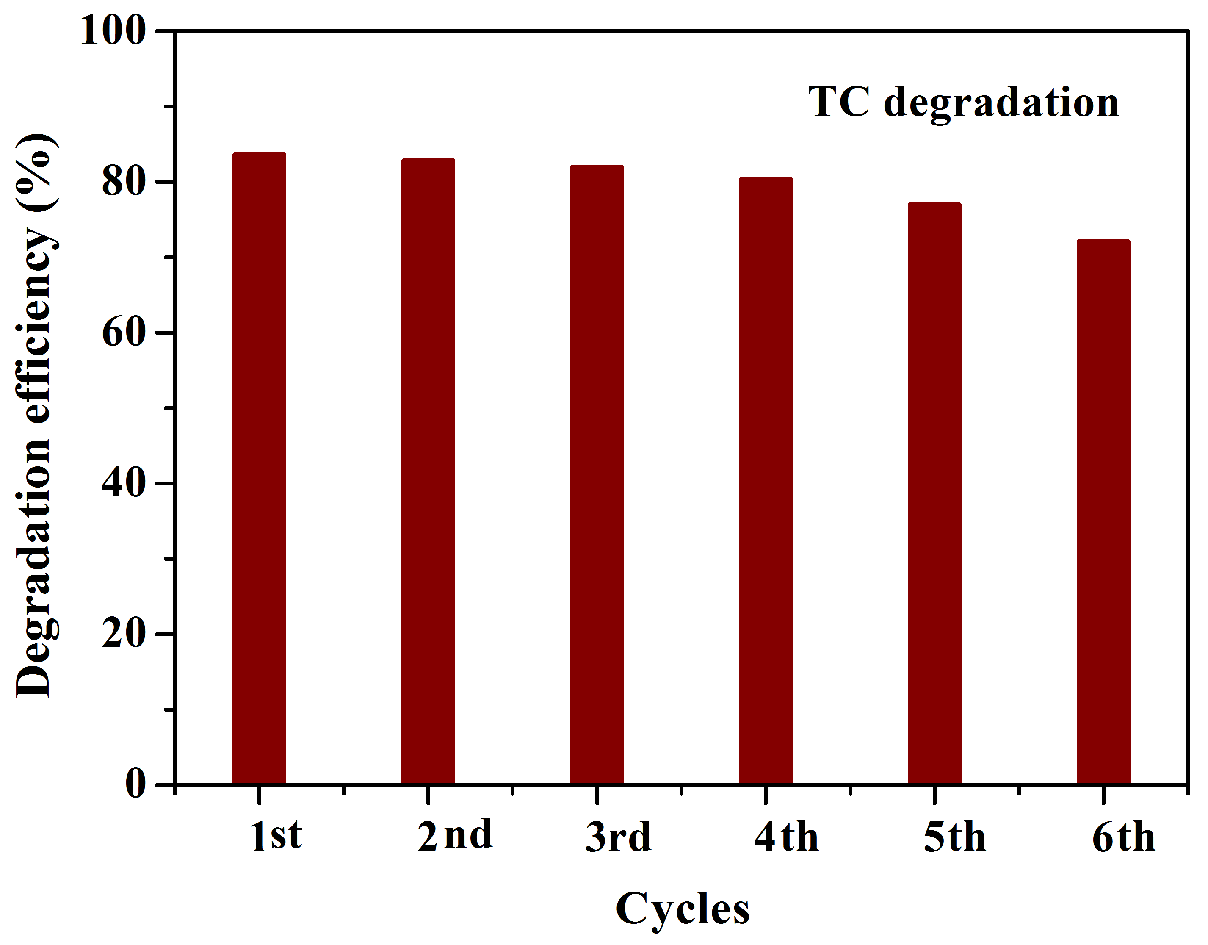


**Figure S3.** The cycling performance of AVO/BOC-10 in the degradation of TC.
